# CircCRIM1 Promotes Hepatocellular Carcinoma Proliferation and Angiogenesis by Sponging miR-378a-3p and Regulating SKP2 Expression

**DOI:** 10.3389/fcell.2021.796686

**Published:** 2021-11-12

**Authors:** Yang Ji, Shikun Yang, Xueqi Yan, Li Zhu, Wenjie Yang, Xinchen Yang, Fei Yu, Longqing Shi, Xi Zhu, Yunjie Lu, Chuanyong Zhang, Hao Lu, Feng Zhang

**Affiliations:** ^1^ Key Laboratory of Liver Transplantation, Hepatobiliary/Liver Transplantation Center, Chinese Academy of Medical Sciences, The First Affiliated Hospital of Nanjing Medical University, Nanjing, China; ^2^ Department of Oncology, The First Affiliated Hospital of Nanjing Medical University, Nanjing, China; ^3^ Department of Hepatobiliary Surgery, The Third Hospital Affiliated to Soochow University, Changzhou, China; ^4^ Department of Infectious Disease, The First People’s Hospital of Kunshan Affliated with Jiangsu University, Zhenjiang, China

**Keywords:** circCRIM1, HCC, MiR-378a-3p, Skp2, proliferation, angiogenesis

## Abstract

Mounting evidence has demonstrated that circular RNAs have an important function in tumorigenesis and cancer evolvement. CircCRIM1 has been shown to be a poor prognostic element in multiple human malignancies. However, the clinical significance and mechanism of circCRIM1 in hepatocellular carcinoma (HCC) is still unclear. The present study confirmed the expression level of circCRIM1 using quantitative real-time PCR. In addition, circCRIM1 siRNA and overexpression vectors were used for transfection into LM3 or Huh7 cells to down- or up-regulate the expression of circCRIM1. *In vitro* and *in vivo* experiments were performed to explore the function of circCRIM1 in HCC. RNA pull-down, RNA immunoprecipitation, fluorescent *in situ* hybridization, and luciferase reporter assays were conducted to confirm the relationship between miR-378a-3p and circCRIM1 or S-phase kinase-associated protein 2 (SKP2) in HCC. Then, circCRIM1 was up-regulated in HCC and its expression level was significantly associated with poor prognosis and clinicopathologic characteristics. CircCRIM1 enhanced the proliferation and angiogenesis of HCC cells *in vitro* and promoted xenograft growth *in vivo*. Moreover, circCRIM1 upregulated the expression of SKP2 by functioning as a sponge for miR-378a-3p. These findings suggest that circCRIM1 boosts the HCC progression via the miR-378-3p/SKP2 axis and may act as a crucial epigenetic therapeutic molecule target in HCC.

## Introduction

Primary hepatocellular carcinoma (HCC) is one of the most common cancers and its mortality ranks third (Sung et al., 2021). Even though both hepatectomy and liver transplantation are the most beneficial treatments for HCC, the overall survival (OS) in HCC patients is still low because of neoplasm recurrence and metastasis with the blood after surgery (Cao et al., 2020). Moreover, the molecular pathogenesis of HCC remains largely unknown and requires further investigation. Therefore, efforts are being made to identify new molecular targets that can be used to improve HCC treatments (Allemani et al., 2018).

Circular RNA (circRNA) is a single-stranded closed loop without the 5′ caps and 3′ poly (A) tails (Kristensen et al., 2019), which is also a part of non-coding RNAs. This structure results in high stability in tissues. CircRNAs are expressed in tissues of many cancers, such as colorectal, liver, and lung cancer. They also adjust gene expression in the human body (Wu et al., 2019; Li Z. et al., 2020; Hu et al., 2020; Zuo et al., 2021). In addition, circRNAs can sponge miRNAs during post-transcriptional regulation (Poliseno et al., 2010; Gu et al., 2021). These findings indicate that circRNAs may serve as potential therapeutic target for cancer.

CircCRIM1 has been identified as a relative factor in multiple human cancer prognoses. In nasopharyngeal carcinoma, circCRIM1 promotes the proliferation, invasion, metastasis, and chemoresistance by sponging miR-422a (Hong et al., 2020). Another study has reported that circCRIM1 is a tumor-positive factor that accelerates the progression and facilitates osteosarcoma autophagy by targeting miR-432-5p (Liu J. et al., 2020). In addition, circCRIM1 has also been reported to be a suppressive factor that inhibits the progression of lung adenocarcinoma by interacting with miR-182 and miR-93 (Wang et al., 2019). However, not many research studies have investigated circCRIM1 in HCC.

S-phase kinase-associated protein 2 (SKP2) is a RING-finger type ubiquitin ligase, a member of cullin-RING ubiquitin ligases (CRLs), and a functional component of ligase complex Skpl-Cullin-F-box (SCF). SKP2 functions as a direct regulator of cyclin-dependent kinases (CDKs) (Lin et al., 2019). In colorectal carcinoma, CDKs interacting with SKP2 contributed to the loss of tumor differentiation and decreased the OS (Shapira et al., 2005). SKP2 also plays an oncogenic role in other human cancers. SKP2 induces tumorigenesis via Hippo signaling (Zhang et al., 2017) in HCC, and promotes tumor progression in breast cancer via PDCD4 ubiquitination (Li et al., 2019). Previous studies have reported that SKP2 induces the proliferation of vascular smooth muscle cells by decreasing P27/P21 expression (Song et al., 2011).

We have previously determined the expression of circCRIM1 in HCC cells, tissues, and adjacent tissues. The data indicated that circCRIM1 has a high expression in HCC cells and tissues, which obviously correlated with poor prognosis and clinicopathological characteristics of patients with HCC, containing tumor size, TNM stage, and Edmondson grade. Moreover, circCRIM1 significantly promoted the progression of HCC via sponging miR-378a-3p and targeting SKP2.Therefore, circCRIM1 can be regarded as a prognostic biomarker and a possible therapeutic target for some patients with HCC.

## Materials and Methods

### Tissues and Cell Lines

A total of 76 pairs of HCC tissues and corresponding adjacent normal liver tissues were obtained from the First Affiliated Hospital in Nanjing Medical University. All samples were retrieved from patients during the operation. Patients receiving anti-tumor therapy were excluded. This research was authorized by the ethics committee of our hospital. HCC cell lines were purchased from the Cell Bank of Type Culture Collection (Shanghai, China). All cell lines were cultured in Dulbecco’s modified Eagle’s medium with 10% fetal bovine serum (Gibco, NY, United States) at 37°C with 5% CO_2_.

### QRT-PCR

TRIzol reagent (Invitrogen, United States) was employed to extract RNA from HCC tissues and cell lines described above. Then, RNA from each specimen was used for reverse transcription of cDNA. The cDNA acting as a template was used for RT-qPCR. The expression of human GAPDH genes was used for normalizing the expression of circRNA or mRNA, while U6 was used for miRNA. Gene expression level was calculated using the 2^−ΔΔCt^ method. The primer sequences used in this study are listed in Supplementary Table S1.

### Nuclear Cytoplasmic Fractionation, RNase R, and Actinomycin D Treatment

Nuclear and cytoplasmic fractions were separated using the PARIS™ kit (Invitrogen, United States). Total RNA (2 µg) and 3 U/µg RNase R (Li S. et al., 2020) (Epicentre Technologies, Madison, United States) were incubated together for 15 min at 37°C. Then, 5 μg/ml actinomycin D (Chen et al., 2020)was introduced to detect the expression of endogenous circRNA and mRNA. CircCRIM1 and linear CRIM1 expression was analyzed by qRT-PCR.

### RNA FISH

To determine the circCRIM1 and miR-378a-3p locations in HCC cells and tissues, FAM-labelled circCRIM1 probes and cy3-labelled miR-378a-3p probes were designed and constructed by Sevicebio (Wuhan, China). Samples were analyzed using a Nikon inverted fluorescence microscope. The respective circCRIM1 and miR-378a-3p probe sequences used in the FISH assay were as follows: 5′-AGTCCAGTT CTCATCTTGTTG GCAAAGTAC-3′ and 5′-CCT​TCT​GAC​TCC​AAG​TCC​AGT-3’.

### Transfection Experiment

Specific circCRIM1 siRNA, miR-378a-3p inhibitors, mimics, and their corresponding NCs were constructed by TSINGKE Biological Technology (Beijing, China). Full-length circCRIM1 was added into the pEX-3 (TSINGKE Biological Technology, Beijing, China) overexpression vector. LM3 and Huh7 cells were transfected with circCRIM1 siRNAs, miRNA-378a-3p mimics, or inhibitors using Lipofectamine 3000 (Invitrogen), while circCRIM1 overexpression plasmids were prepared using jetPRIME (Polyplus-transfection, France). Cells were harvested after a 48-h transfection. SKP2-overexpressing lentivirus and SKP2 shRNAs were constructed by GenePharma (Shanghai, China) and transfected. All of the sequences used in the present study are listed in Supplementary Table S1.

### CCK-8 Assay

For detecting cell proliferation, we used CCK-8 assay kit (Dojindo, Japan). Briefly, a 96-well plate were seeded in (1.0 × 10^3 cells/well) and were cultured 24, 48, 96, 120 and 148 h. At the certain point. 10μL of CCK-8 assay solution spread in each well was incubated for 120min in the dark. An enzyme immunoassay analyzer (Thermo Fisher Scientific, Inc. Waltham, MA, United States) was used to evaluate the absorbance of each well at OD450 nm.

### Clone Formation Assay

The stably transfected Huh7 and LM3 cells were plated in six-well plates (800 cells/well). The plates cultured in DMEM with 10% fetal bovine serum (FBS) were harvested after 2 weeks. Then, the colonies were fixed with 1 ml of 4% paraformaldehyde (Sevicebio, Wuhan, China) for 25 min and stained with 0.1% crystal violet (Beyotime). The colonies were photographed and counted after washing with phosphate-buffered saline (PBS).

### EdU Assay

After seeded evenly in 24-well plates, HCC cells were cultured with DMEM. Subsequently, Edu solution (Beyotime) was added in each wells and incubated for 2 h. The rest of steps were following the manufacturer’s instruction. The specimens were imaged and measured by a microscope (Olympus, Tokyo, Japan).

### Western Blot Analysis

Protease and phosphorylation inhibitor cocktail (New cell & Molecular Biotech Suzhou, China) in the RIPA buffer was used to lyse Huh7 and LM3 cells. Then, the total protein was separated via SDS-PAGE and transferred onto a PVDF membrane (Millipore, United States). Next, the membranes were blocked using Quick Block™ Blocking Buffer for Western Blot (Beyotime, Shanghai, China) within 15 min. After washing with TBST, the membranes were incubated with the appropriate primary antibodies. After an incubation with the corresponding HRP-labelled secondary antibodies on the next day, an enhanced chemiluminescence detection system was used for antigen-antibody complex visualization. All antibodies used in the study are listed in Supplementary Table S2.

### Flow Cytometry Assay

For flow cytometry assay, 1 × 10^6^ cells were suspended in 1 ml of PBS at room temperature. Then, 3 ml of precooled absolute ethyl alcohol were added and the mixture was kept overnight at −20°C. On the next day, the fixed cells were centrifuged and hydrated using PBS for 15 min, centrifuged again, then stained with 1 ml of the DNA staining solution (MultiSciences Biotech Co., Ltd., Hangzhou, China) away from light for at least 30 min. Finally, flow cytometry (LSR, BD Biosciences) was used to analyze the cell cycle.

### HUVEC Tube Formation Assays

The conditioned medium was obtained from the DMEM cultured with LM3 or Huh7 cells transfected with circCRIM1-overexpressing lentivirus or si-RNA for 48 h. Subsequently, 50 µL of precooled Matrigel (BD Biosciences, San Jose, CA, United States) were carefully added into precooled 96-well plates for 30 min at 37°C. Then, HUVECs were seeded and cultured with the conditioned medium for 6 h at 37°C. An inverted microscope (Olympus) was used to observe and photograph tube formation.

### HUVEC Recruitment Assays

First, HUVECs were harvested and suspended in DMEM without FBS. Next, 400 µL of cell suspension containing ten thousand cells were seeded into the upper chambers (Millicell, United States). A total of 600 µL of TCM were added into the lower chambers. After 36 h, the Transwell chambers were fixed with 4% formalin (Sevicebio, Wuhan, China). Then, 0.1% crystal violet was added dye for cell visible for 30 min. After washing twice with PBS, the cells migrated through the membrane were counted and photographed using a microscope (Olympus).

### Biotin-Labelled RNA Pull-Down

The biotinylated circCRIM1 probes were designed by RiboBio (Guangzhou, China). First, 1 × 10^7^ cells were lysed. Next, 50 µL of beads were incubated with 50 µL of circCRIM1 probe for 2 h. After washing twice with 50 µL of Tris, the cell lysates obtained from LM3 cells with probe-coated beads were incubated overnight at 4°C. At last, we eluted and extracted the RNA complexes for qRT-PCR.

### RIP Assay

RNA immunoprecipitation assay (Zhang et al., 2021) was conducted using a Magna RIP RNA-Binding Protein Immunoprecipitation Kit (Millipore, United States) following the manufacturer’s protocol. Purified RNA extracted from cells was used for qRT-PCR.

### Dual-Luciferase Assay

WT and MUT circCRIM1 (pLuc-Firefly-Renilla with circCRIM1 WT or MUT sequence) were constructed by GeneChem Co. (Shanghai, China), while WT and MUT SKP2 were constructed. Lipofectamine 3000 was used to transfect Huh7 and LM3 cells with the corresponding reporter plasmids and microRNA mimics or the negative control. Finally, their activity was detected using the Dual-Luciferase Reporter System Kit (E1910, Promega, United States). Each experiment was repeated three times.

### Animal Experiments

For tumor growth *in vivo*, four-week-old nude female mice were purchased from the institution: Animal Core Facility of Nanjing Medical University (Nanjing, China). Then, those mice were randomly divided into four groups (*n* = 5 for each group). After, 2 × 10^6 Huh7 cells were suspended in 100uL PBS for each mouse and subcutaneously injected into the dorsal flank, which were stably down-regulated the expression of circCRIM1 or NC. The rest of the mice were injected with LM3 cells containing circCRIM1-overexpressing plasmids or NC. Subsequently, tumor volumes and weights were measured at several time points. All samples were fixed in 4% paraformaldehyde and stained using IHC.

### Immunohistochemistry

For IHC, the xenografts and HCC tissues were dewaxed and rehydrated with a gradient of ethanol. After an incubation in citrate buffer (10 mM, pH 6.0) for 20 min at 95°C, the samples were first incubated with polyclonal antibodies against SKP2 (Proteintech) and CD34 (Cell Signaling Technology) overnight at 4°C and then with secondary antibodies for 1 h. The staining was scored in terms of the rate of positive cells. Intensity of each sample was measured using four grades: 0, negative; 1, weak; 2, moderate; and 3, strong.

### Statistical Analysis

All data were analyzed using SPSS 24.0 (SPSS, Chicago, IL, United States) and Prism 8 (GraphPad Software, La Jolla, CA, United States). Differences between the means were analyzed using Student’s t-test. The correlations between cricCRIM1 and miR-378a-3p or miR-378a-3p and SKP2 expression levels were analyzed using Pearson’s test (r, P). The χ2 test was used to evaluate the association between circCRIM1, miR378a-3p, SKP2 expression, and clinicopathological parameters. The OS rate was calculated using the Kaplan-Meier method and compared with a log-rank test. *p < 0.05, **p < 0.01, and ***p < 0.001 were considered as statistically significant.

## Results

### Upregulation of circCRIM1 in Both HCC Cell Lines and Tissues Is Associated With Poor Prognosis

**FIGURE 1:**
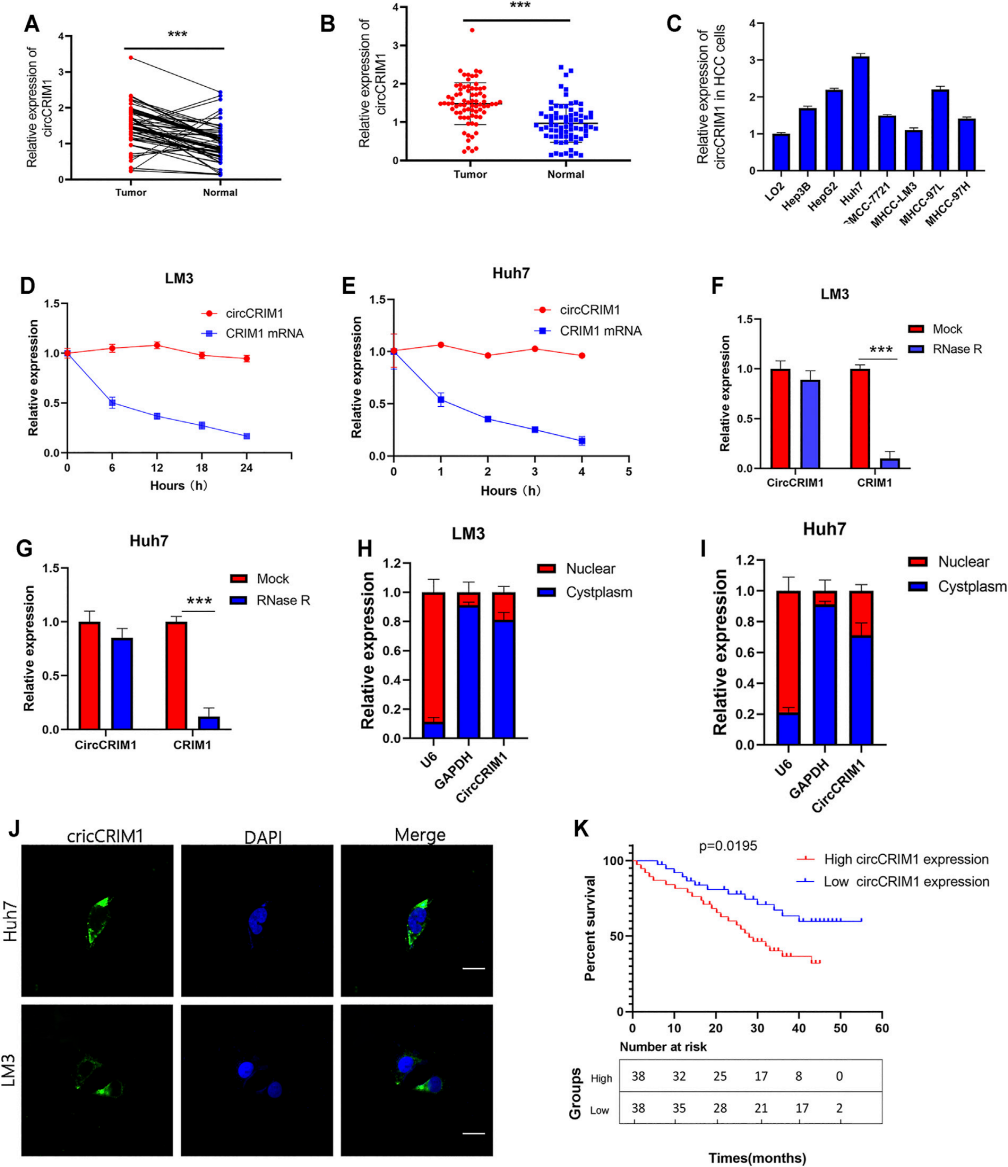
CircCRIM1 is upregulated in HCC cell lines and tissues. **(A,B)** Relative circCRIM1 expression in HCC tissues (tumor) and adjacent non-tumor tissues (adjacent) was detected by qRT-PCR (*n* = 76). **(C)** Relative circCRIM1 expression in HCC cell lines was determined by qRT-PCR. **(D,E)** Actinomycin D treatment was used to evaluate the stability of circCRIM1 and CRIM1 mRNA in HCC cells. **(F,G)** CircCRIM1 and linear CRIM1 expression in HCC cells was detected after RNase R treatment and compared with mock treatment. **(H,I)** Nuclear-cytoplasmic fractionation assay results indicated that cirCRIM1 is primarily localized in the cytoplasm of HCC cells. GADPH and U6 genes were used as cytoplasmic and nuclear controls, respectively. **(J)** FISH results showed cellular localization of circCRIM1. The circCRIM1 probe was labeled with FAM (green), while nuclei were stained with DAPI (blue).Scale bar = 10 μm. **(K)** Kaplan-Meier survival analysis showed that HCC patients with high circCRIM1 expression had a lower OS (*p* = 0.0195) than those with low circCRIM1 expression. All data are presented as the means ± SD of three independent experiments. *p < 0.05, **p < 0.01, and ***p < 0.001.

The expression of circCRIM1 in HCC and adjacent normal tissue samples from 76 patients was detected by qRT-PCR. CircCRIM1 expression in HCC tissues was higher than that in corresponding adjacent normal samples (Figures 1A,B). In addition, circCRIM1 was upregulated in human HepG2, Hep3B, SMMC-7721, MHCC-LM3, MHCC-97L, MHCC-97H, and Huh-7 cell lines (HCC cell lines) compared with normal hepatic cells L02 (Figure 1C), indicating that circCRIM1 expression levels may becorrelated with HCC progression. Based on the circBase annotation (http://www.circbase.org/), hsa_circ_0002346 (chr2: 36623756–36,669,878) (also named circCRIM1) originated from the 2, 3, and 4 exons in the CRIM1 gene. In order to determine the circular and linear forms of CRIM1, we designed divergent and convergent primers, respectively. Actinomycin D (Chen et al., 2020) assays and RNase R (Li S. et al., 2020) assays showed that circCRIM1 was more stable than linear CRIM1 (Figures 1D–G). In addition, the results of FISH assay were identified that circCRIM1 was mainly located in the cytoplasm (Figure 1H). Nuclear cytoplasmic fractionation had similar results (Figures 1I,J). The samples were split into two groups to investigate the connection between circCRIM1 and to investigate the clinical significance of HCC. The high circCRIM1 expression group was associated with tumor size (*p* = 0.0049), TNM stage (*p* = 0.0216), as well as Edmondson grade (*p* = 0.0280), rather than gender, age, liver cirrhosis, AFP, or HBsAg (*p* > 0.05; Table 1). In addition, HCC patients with higher circCRIM1 expression had a conspicuously worse OS in our center (Figure 1K). Taken together, circCRIM1 is a stable existing circRNA located in the cytoplasm that has the potential to be a biomarker for the diagnosis and prognosis of HCC.

**TABLE 1:** Correlation between circCRIM1 expression and clinicopathological features in HCC tissues (*n* = 76, χ^2^-test).

	CircCRIM1 expression		
	High	Low	
Variable	38	38	*p*-value
Age (year)	—	—	0.2119
<60	29	24	—
≥60	9	14	—
Gender	—	—	0.4721
Male	23	26	—
Female	15	12	—
Tumor size	—	—	0.0116*
<5 cm	14	25	—
≥5 cm	24	13	—
TNM stage	—	—	0.0216*
I–II	25	15	—
III–IV	13	23	—
Liver cirrhosis	—	—	0.2432
Yes	25	20	—
No	13	18	—
AFP (ng/ml)	—	—	0.6445
≤200	18	16	—
>200	20	22	—
HBsAg	—	—	0.9999
Positive	28	28	—
Negative	10	10	—
Edmondson grade	—	—	0.0280*
I–II	30	21	—
III–IV	8	17	—
*p* < 0.05	—	—	—

### CircCRIM1 Accelerates Proliferation of HCC Cells and Promotes Transition From G1 to S Phase

**FIGURE 2:**
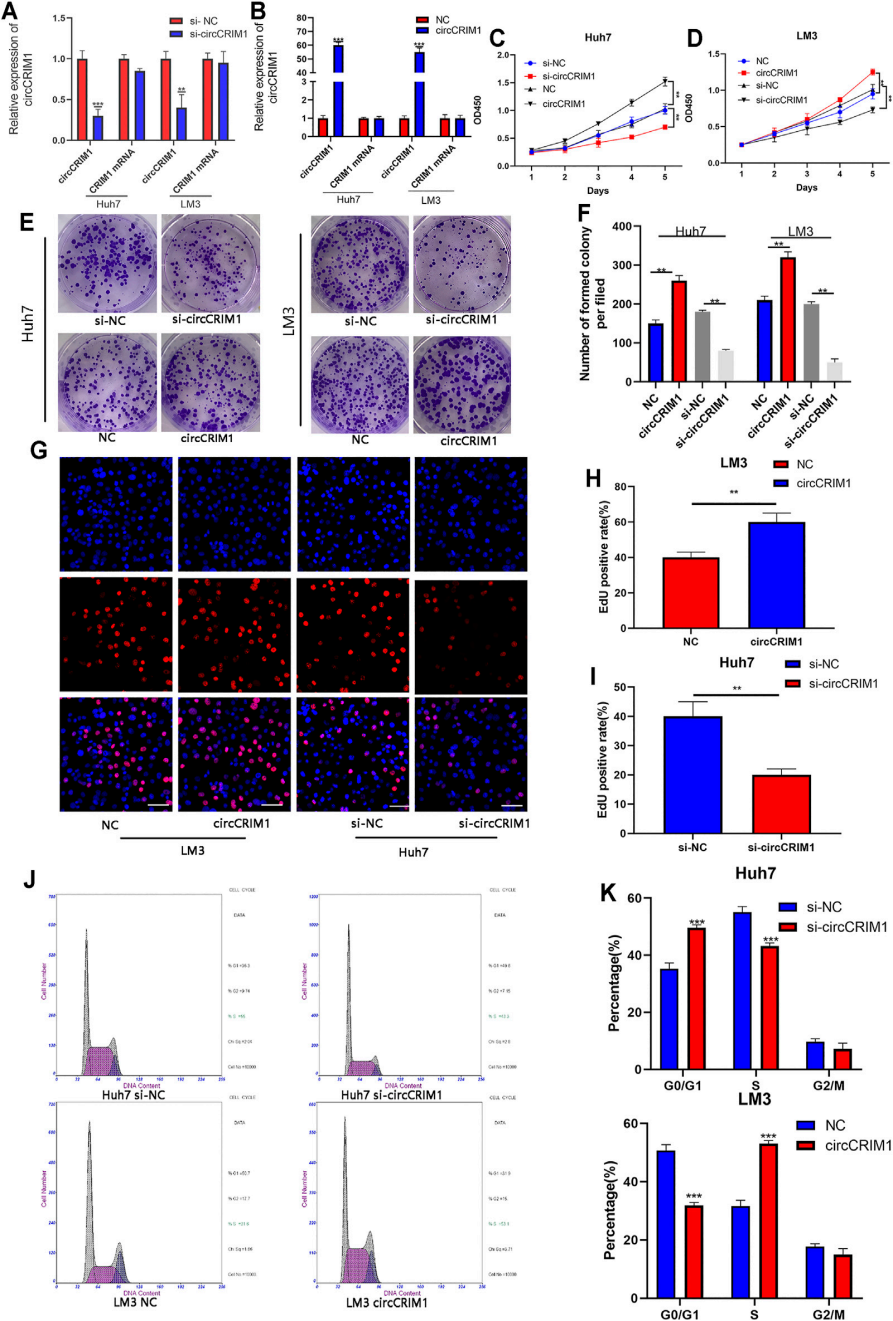
CircCRIM1 promotes the proliferation of HCC cells and accelerates the transition from G1 to S phase. **(A,B)** QRT-PCR analysis of circCRIM1 and CRIM1 mRNA expression in LM3 and Huh7 cells transfected with si-NC, si-circCRIM1, circCRIM1, and NC. **(C,D)** Cellular growth curves were evaluated by CCK-8 assays after knocking down or overexpressing circCRIM1 in LM3 or Huh7 cells. **(E,F)** Colony formation assays were performed to evaluate cell proliferation. **(G–I)** EdU assays were performed on HCC cells to evaluate cell proliferation. Scale bar, 50 μm. **(J,K)** Flow cytometry assays show that circCRIM1 can accelerate the G1 to S phase cell cycle transition, while circCRIM1 knockdown increases the percentage of G1 phase cells. All data are presented as the means ± SD of three independent experiments. *p < 0.05, **p < 0.01, and ***p < 0.001.

To evaluate the biological function of circCRIM1 in the progression of HCC, a short interfering RNA (siRNA) targeting the back splice region of circCRIM1 was transfected into HCC cells to knock down circCRIM1. In addition, circCRIM1 plasmids were transfected into Huh7 and LM3 cell lines to overexpress circCRIM1 without changing the expression of CRIM1 mRNA. Then, qRT-PCR was conducted to verify the efficiency and specificity of knocked down and overexpressed circCRIM1 in Huh7 and LM3 cells (Figures 2A,B). Cell Counting Kit-8 (CCK-8) assays were also conducted. Their results suggested that circCRIM1 silencing notably inhibits the proliferation of Huh7 cells, while circCRIM1 overexpression increases the growth of LM3 cells (Figures 2C,D). Concordant with these results, colony formation assays showed that downregulating circCRIM1 expression curbed cell growth. Conversely, overexpressing circCRIM1 increased the colony forming ability of LM3 cells (Figures 2E,F). In addition, 5-ethynyl-2-deoxyuridine (EdU) assays showed that the growth in siRNA-transfected cells was inhibited at first and subsequently promoted by circCRIM1 overexpression (Figures 2G–I). Then, the cell cycle assay revealed that silencing circCRIM1 arrested cells in the G0/G1 phase. Conversely, circCRIM1 overexpression induced the transition to the S phase (Figures 2J,K). Therefore, circCRIM1 exerts oncogenic effects on HCC cells.

### CircCRIM1 Enhances Angiogenic Capability of HCC Cells

**FIGURE 3:**
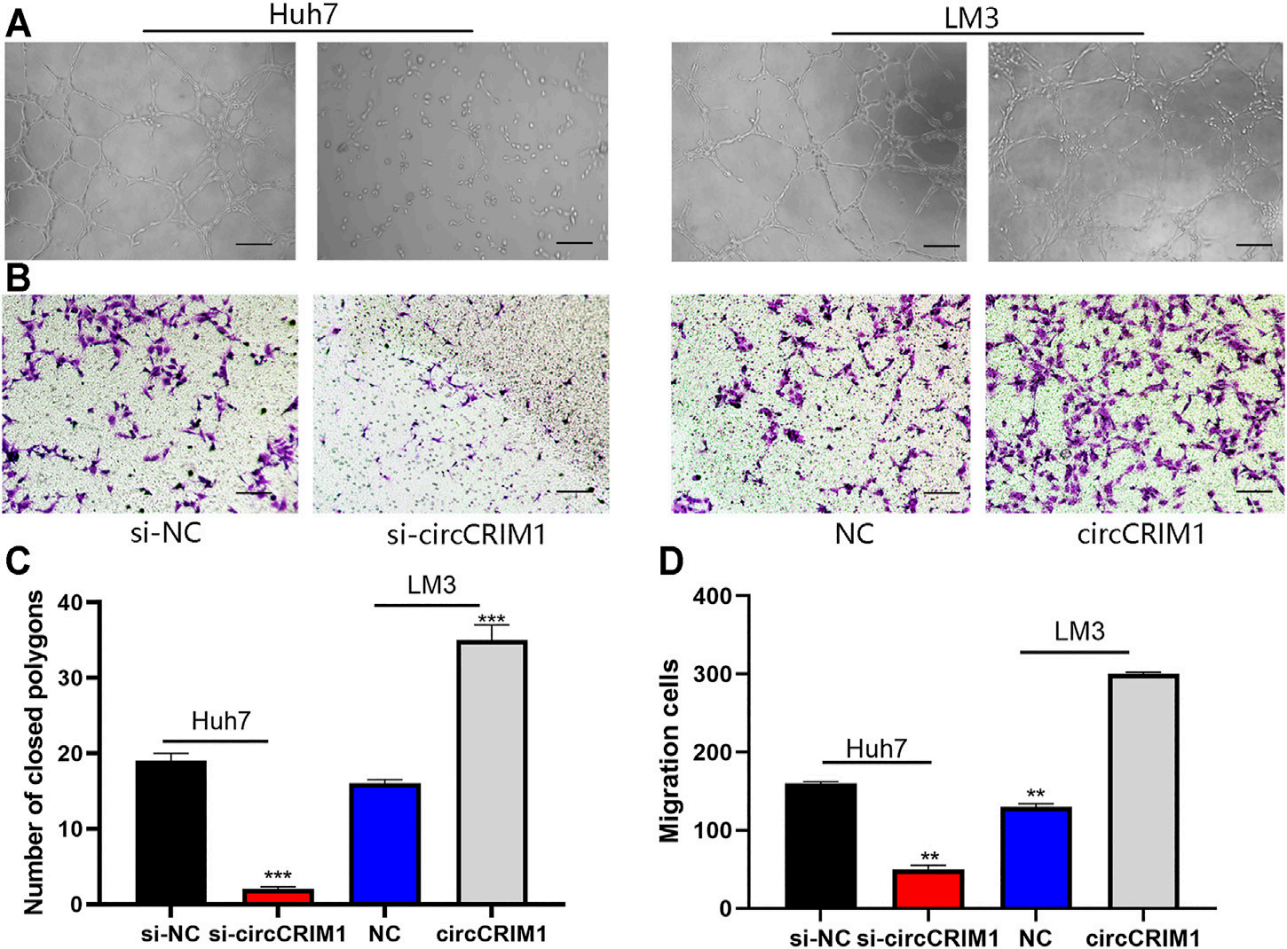
CircCRIM1 enhances the angiogenic capability of HCC cells. **(A,C)** Tube formation assays with HUVECs in indicated conditioned media. Numbers of branches were calculated using Image Pro Plus 6. Scale bar, 50 μm. **(B,D)** Endothelial recruitment assays with HUVECs performed for each group. Scale bar, 50 μm Cell migration was quantified using cell numbers. All experiments were performed three times. Data are mean ± SD. *p < 0.05, **p < 0.01, and ***p < 0.001.

Angiogenesis is essential for tumor cell proliferation. In order to study the functions of circCRIM1 in HCC angiogenesis, different tumor-conditioned media (TCM) were used to perform endothelial recruitment and tube formation assays *in vitro*. Compared with the control group, human umbilical cord endothelial cell (HUVEC) tube formation was decreased in the si-cricCRIM1 group (Figures 3A,C). The capability of LM3 cells to accelerate HUVEC tube formation was boosted in the circCRIM1 overexpression group (Figures 3A,C). Next, the function of cricCRIM1 in HUVEC migration was investigated using endothelial recruitment assays. Compared with the control group, the extent of HUVEC migration was enhanced by TCM, which was collected from LM3 cells transfected with circCRIM1-overexpressing plasmids (Figures 3B,D). On the contrary, TCM originating from si-circCRIM1 Huh7 cells significantly decreased HUVEC migration (Figures 3B,D). Taken together, these data demonstrate that circCRIM1 enhances the capacity of cancer cells to accelerate HUVEC migration and tube formation.

### CircCRIM1 Functions as miR-378a-3p Sponge in HCC

**FIGURE 4:**
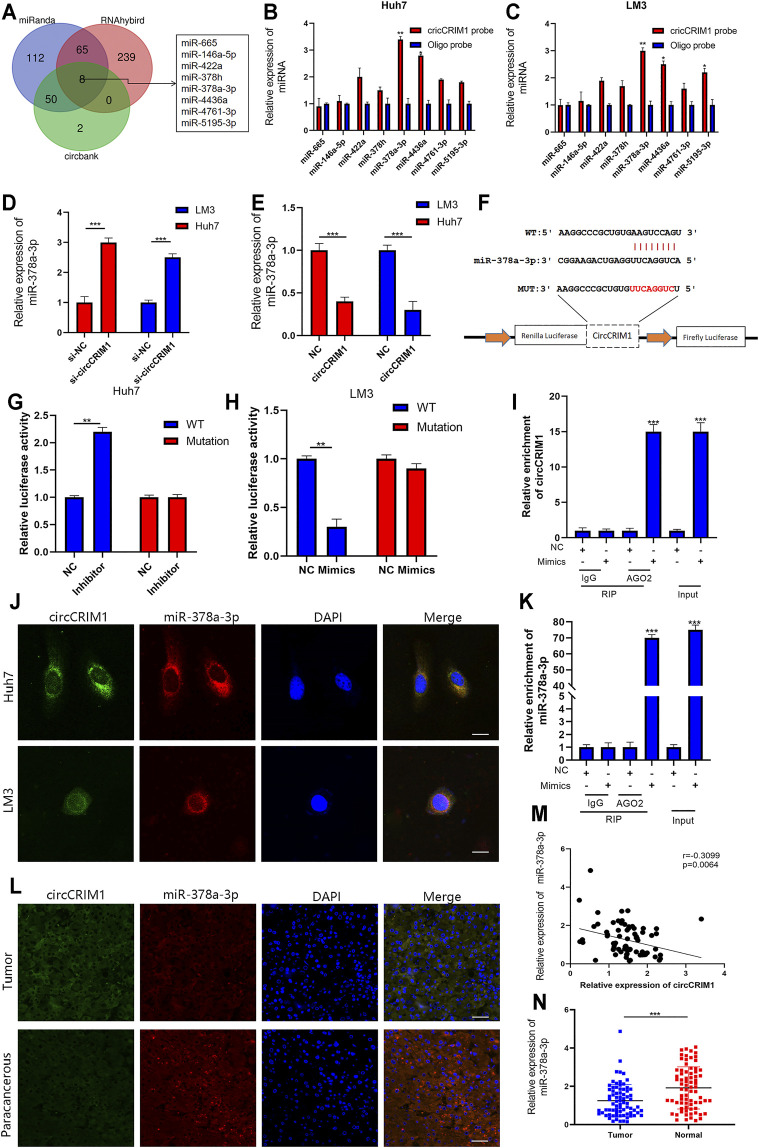
CircCRIM1 functions as miR-378a-3p sponge in HCC **(A)** Venn diagram showing the overlap of target miRNAs for circCRIM1 predicted by miRanda, circbank, and RNAhybrid. **(B,C)** Relative abundance of eight miRNA candidates in Huh7 and LM3 lysates with circCRIM1 or oligo probe was examined by qRT-PCR. **(D,E)** Relative levels of miR-378a-3p in Huh7 and LM3 cell lines transfected with si-circCRIM1, si-NC, circCRIM1, or NC were detected by qRT-PCR **(F)** Schematic of wild-type (WT) and mutant (MUT) circCRIM1 luciferase reporter vectors **(G,H)** Effects of miR-378a-3p mimics, inhibitor, and NC on luciferase activity were detected in HCC cells transfected with luciferase reporter vectors expressing WT, MUT, or NC. **(I,J)** RIP assay for circCRIM1 was performed with anti-AGO2 antibody in HCC cells transfected with mimics or NC, and expression of circCRIM1 and miR-378a-3p was detected by qRT-PCR. **(K)** Co-localization of circCRIM1 and miR-378a-3p in HCC cells was detected using a FISH assay. Scale bar,10 μm **(L)** FISH results showing co-localization of circCRIM1 and miR-378a-3p in HCC and para-cancerous tissues from patients. Scale bar, 25 μm **(M)** Obviously negative correlation between levels of circCRIM1 and miR-378a-3p in 76 pairs of freshly-frozen HCC tissues and matched normal liver tissues was analyzed by Pearson correlation analysis. **(N)** Relative miR-378a-3p expression in 76 pairs of freshly-frozen HCC tissues and matched normal liver tissues. All data are presented as the means ± SD of three independent experiments. *p < 0.05, **p < 0.01, and ***p < 0.001.

Researchers have suggested that circRNAs have the ability to sponge miRNA to regulate gene expression (Wu et al., 2019). Therefore, we analyzed the circCRIM1 sequence using miRanda, circbank, and RNAhybrid (Xia et al., 2009; Krol et al., 2010). Eight candidate miRNAs that may bind to circCRIM1 were identified for further study (Figure 4A). Next, the biotinylated circCRIM1 probe was used for the pull-down assay to indirectly capture binding miRNAs. The fold changes in miR-378a-3p pulled down by the circCRIM1-specific probe were observed in both HCC cell types (Figures 4B,C). Silencing or overexpressing circCRIM1 resulted in fold changes in the microRNA expression in LM3 or Huh7 cells, respectively (Figures 4D,E). To identify the relationship between circCRIM1 and miR-378a-3p, a dual-luciferase reporter assay was carried out. Special luciferase plasmids with cloned full-length circCRIM1 were constructed and transfected into cells with the microRNA mimics or inhibitor. Luciferase reporter genes contained the primary wild-type (WT) or mutant (MUT) circCRIM1 sequence based on the binding site (Figure 4F). When circCRIM1-WT reporter genes and the microRNA mimics were co-transfected into LM3 cells, the result was obviously decreased, while the inhibitor enhanced the luciferase activity of Huh7 cells that were transfected with WT plasmids. However, miR-378a-3p mimics, inhibitor, or corresponding negative control (NC) did not alter the results of luciferase activity after co-transfection with mutant reporters, indicating that circCRIM1 expression has an opposite effect on miR-378a-3p expression (Figures 4G,H). In general, miRNAs bind to Argonaute-2 (AGO2), which has a key role in RISC components (Xia et al., 2009). Thus, an anti-AGO2 RIP assay (Zhang et al., 2021) was carried out. At the same time, the data suggested that both circCRIM1 and miR-378a-3p from LM3 cell lysates were combined via anti-AGO2 antibody, rather than IgG (Figures 4I,J). FISH assays revealed that both of them were more co-localized in the cytoplasm than in the cell nuclei of Huh7 and LM3 cells (Figure 4K). In addition, FISH suggested that circCRIM1 was significantly expressed in the HCC samples, while the miRNA was mostly located in the para-cancerous tissues (Figure 4L). Contrary to circCRIM1, miR-378a-3p had a lower expression in HCC tissues than in liver para-cancerous tissues (Figures 4M,N). The miRNA in HCC tissues had a clinical significance with tumor size (*p* = 0.0029), TNM stage (*p* = 0.0058), and as well as Edmondson grade (*p* = 0.028; Table 2). Moreover, patients with high expression of miR-378a-3p had better OS (Supplementary Figure S1). These data reveal that circCRIM1 functions as a sponge for miR-378a-3p in HCC.

**TABLE 2:** Correlation between miR-378a-3p expression and clinicopathological features in HCC tissues (*n* = 76, χ^2^-test).

	miR-378a-3p expression		
	High	Low	—
Variable	38	38	*p*-value
Age (year)	—	—	0.8028
<60	27	26	—
≥60	11	12	—
Gender	—	—	0.0816
Male	25	24	—
Female	13	14	—
Tumor size	—	—	0.0029*
<5 cm	26	13	—
≥5 cm	12	25	—
TNM stage	—	—	0.0058*
I–II	26	14	—
III–IV	12	24	—
Liver cirrhosis	—	—	0.8154
Yes	22	23	—
No	16	15	—
AFP (ng/ml)	—	—	0.3561
≤200	15	19	—
>200	23	19	—
HBsAg	—	—	0.1181
Positive	25	31	—
Negative	13	7	—
Edmondson grade	—	—	0.0280*
I–II	30	21	—
III–IV	8	17	—
*p* < 0.05	—	—	—

### MiR-378a-3p Attenuates Oncogenic Effects of circCRIM1 in HCC Cells

**FIGURE 5:**
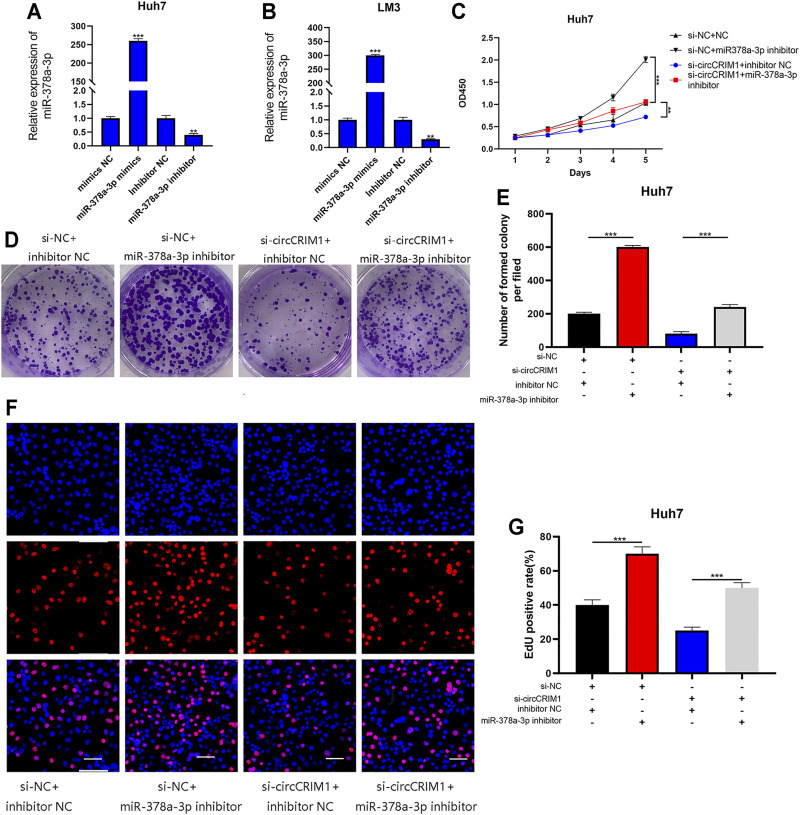
MiR-378a-3p attenuates the oncogenic effects of circCRIM1 in HCC cells. **(A,B)** Expression of miR-378a-3p in LM3 and Huh7 cells transfected with miR-378a-3p mimics, inhibitor, and corresponding NC was detected by qRT-PCR. **(C)** Cellular growth curves were evaluated by CCK-8 assays after transfection with miR-378a-3p inhibitor and NC in Huh7 cells. **(D,E)** Colony formation assays were performed to evaluate cell proliferation. **(F,G)** EdU assays for HCC cells were performed to evaluate cell proliferation. Scale bar, 50 μm. All data are presented as the means ± SD of three independent experiments. *p < 0.05, **p < 0.01, ***p < 0.001, and ****p < 0.0001.

Rescue experiments were implemented with co-transfection of the miRNA mimics or their inhibitors with si-circCRIM1 or circCRIM1 plasmids. QRT-PCR was employed to verify the effects of its mimics and inhibitors in LM3 and Huh7 cells (Figures 5A,B). The results showed that the inhibitors significantly promoted the growth of Huh7 cells, while the inhibitory effect on Huh7 cell proliferation caused by circCRIM1 downregulation was reversed by the introduction of the mentioned inhibitors in CCK-8 assays, colony formation, and EdU assays (Figures 5C–G). Data were also obtained after transfection with mimics and circCRIM1 plasmids in LM3 cells (Supplementary Figure S1). In conclusion, miR-378a-3p plays an anti-oncogenic function in HCC cells and acts as a critical downstream target of circCRIM1.

### SKP2 Is a Direct Target of miR-378a-3p and Indirectly Regulated by circCRIM1

**FIGURE 6:**
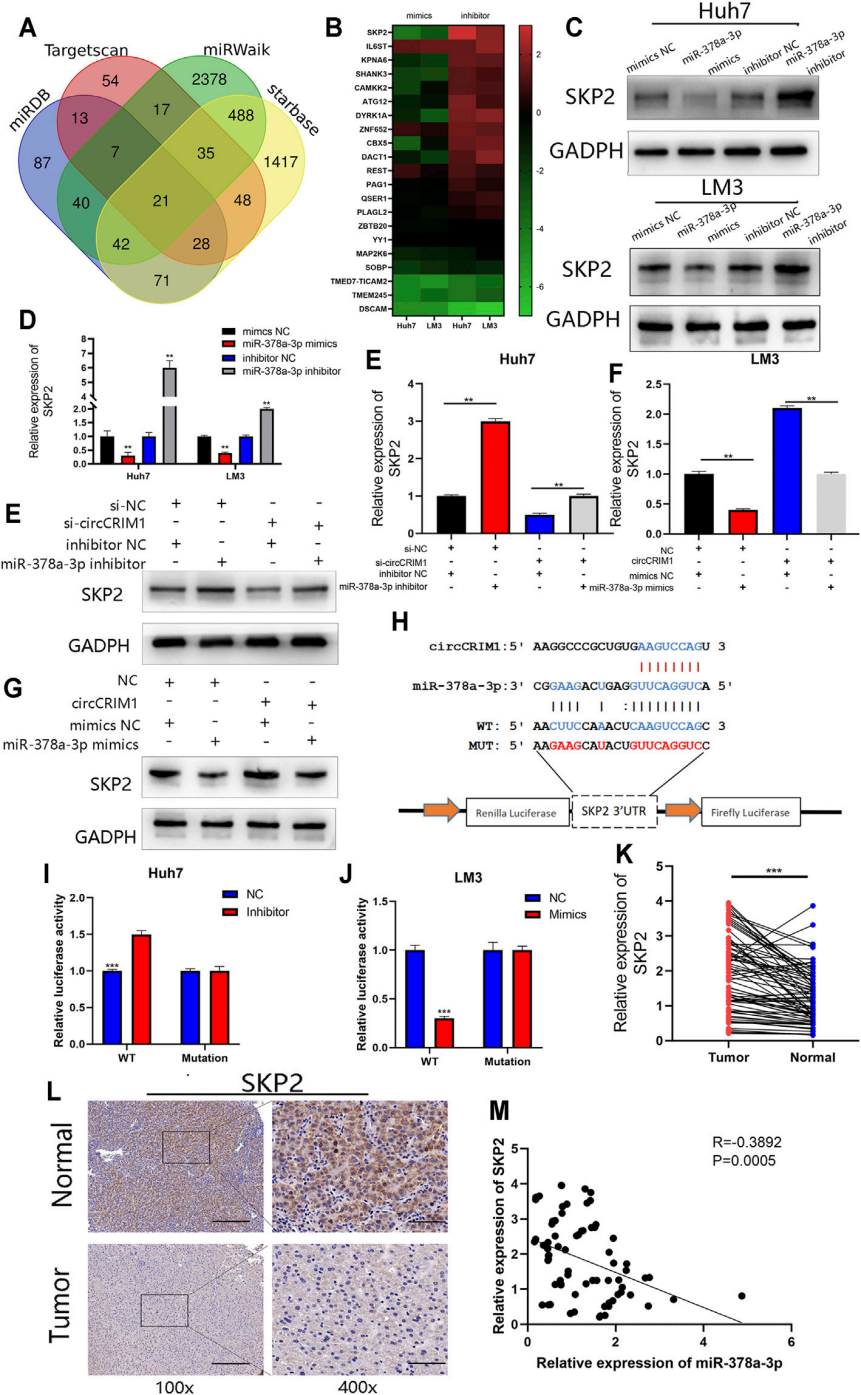
SKP2 is a direct target of miR-378a-3p and is indirectly regulated by circCRIM1. **(A)** Venn diagram showing 21 genes that are putative miR-378a-3p targets predicted by four algorithms (TargetScan, miRDB, starbase, and miRWalk). **(B)** Heat map was used to visualize the expression of predicted target genes in LM3 and Huh7 cells transfected with miR-378a-3p mimics, inhibitor, and corresponding NC. **(C,D)** SKP2 expression in Huh7 and LM3 cells was analyzed by RT-qPCR and western blotting after transfection with miR-378a-3p mimics, inhibitor, and corresponding NC. **(E–G)** SKP2 expression was analyzed by RT-qPCR and western blotting. LM3 cells were transfected with miR-378a-3p mimic or co-transfected with corresponding indicated circCRIM1 vectors and Huh7. **(H)** Schematic of SKP2 3′UTR WT and MUT luciferase reporter vectors. **(I,J)** Effects of miR-378a-3p mimics and inhibitor on luciferase activities of SKP2 3′UTR WT and MUT. **(K)** RT-qPCR analysis of SKP2 expression in HCC tissues (*n* = 76) paired with adjacent normal tissues (*n* = 76). **(L)** IHC staining of SKP2 in HCC and adjacent normal tissues from patients. Samples were imaged at 100× and 400× magnification. Scale bar, 100 and 25 μm **(M)** Negative relationship between levels of miR-378a-3p and SKP2 was identified in 76 paired HCC and normal liver tissues by Pearson correlation analysis. All data are presented as the means ± SD of three independent experiments. *p < 0.05, **p < 0.01, ***p < 0.001, and ****p < 0.0001.

To investigate the regulatory molecule mechanism of miR-378a-3p in HCC, four online databases (TargetScan, miRDB, Starbase, and miRWalk) were used to search for its candidate target genes (Xia et al., 2009; Li J.-H. et al., 2014; Wong and Wang, 2015; Sticht et al., 2018). A total of 21 genes were filtered out from the four databases by overlapping the prediction results for miR-378a-3p (Figure 6A). QRT-PCR experiments were conducted to detect the expression of these genes in Huh7 and LM3 cells with the miRNA mimics or inhibitors. The results suggested that SKP2 was the most likely target (Figure 6B). In addition, miR-378a-3p silencing up-regulated the expression of SKP2 in HCC cells, while its mimics transfected into HCC cells obviously decreased the mRNA and protein levels of SKP2 (Figures 6C,D). Moreover, SKP2 expression was up-regulated with circCRIM1 overexpression, while co-transfection with miR-378a-3p mimics recovered SKP2 expression in LM3 cells. At the same time, Huh7 cells were co-transfected with si-circCRIM1 and the microRNA inhibitors. The depletion of miR-378a-3p rescued the abundance of SKP2, which was down-regulated by si-circCRIM1 (Figures 6E–G). A luciferase reporter gene assay was then performed to further evaluate the connection between SKP2 mRNA and miR-378a-3p. In addition, luciferase reporter plasmids containing WT SKP2 3′-UTRs and MUT SKP2 3′-UTRs were constructed (Figure 6H). The WT plasmids’ luciferase activity was significantly inhibited by the miRNA mimics and increased by the inhibitors. Conversely, the MUT plasmid co-transfection with miR-378a-3p mimics or inhibitors exhibited no evident differences (Figures 6I,J). The SKP2 expression levels of HCC tissues were negatively connected with miR-378a-3p (Figure 6M). In addition, immunohistochemistry (IHC) and qRT-PCR assays further confirmed that SKP2 was higher in HCC samples than in adjacent normal samples (Figure 6K,L). Meanwhile, HCC patients in the high SKP2 expression group had a worse OS (Figure. S2F). These results suggest that circCRIM1 up-regulates SKP2 by interacting with miR-378a-3p in HCC.

### CircCRIM1 Enhances HCC Proliferation and Angiogenesis via miR-378a-3p/SKP2 Axis

**FIGURE 7:**
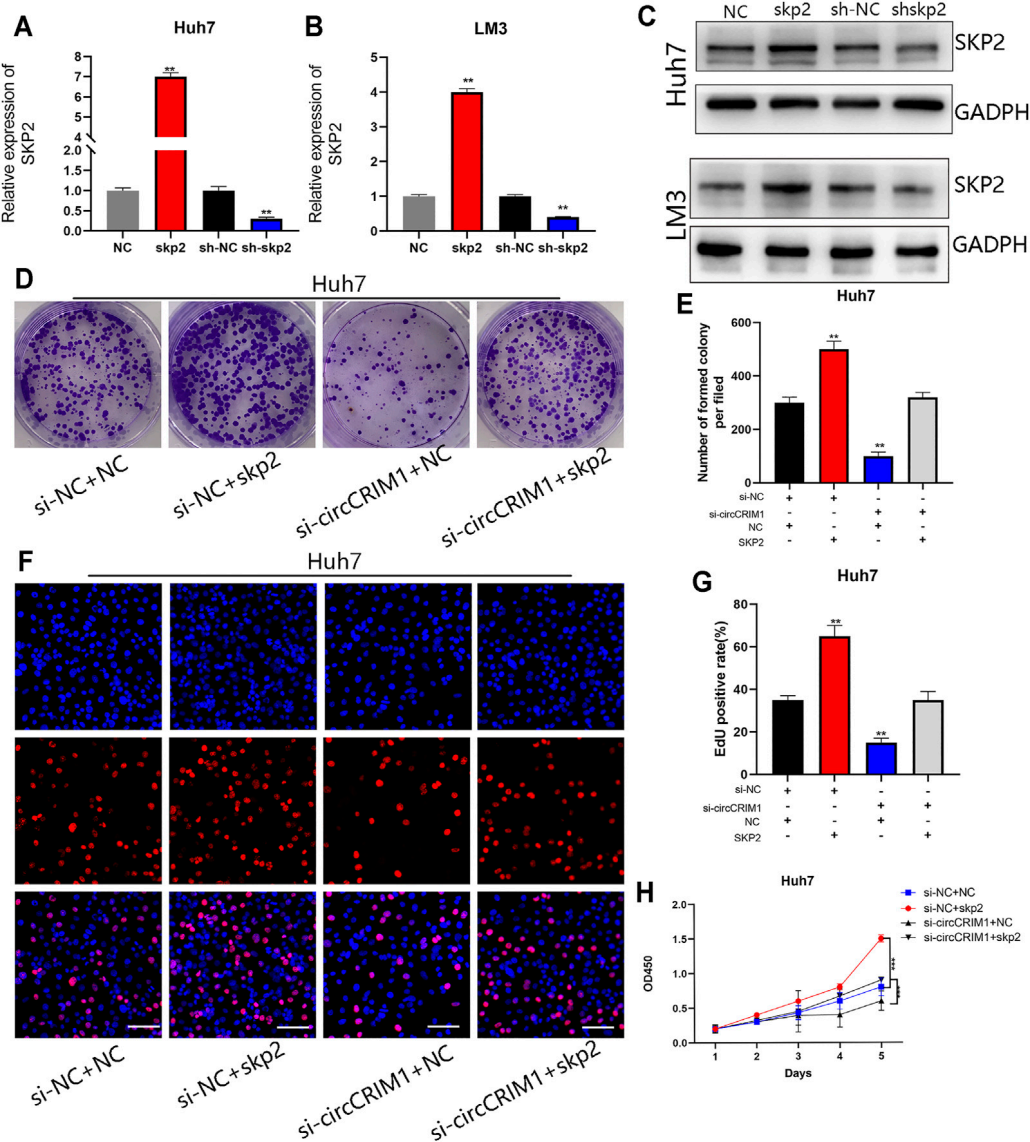
CircCRIM1 promotes HCC proliferation and angiogenesis via miR-378a-3p/SKP2 axis. **(A–C)** Transfection efficiency of shSKP2, shNC, SKP2, and NC in LM3 and Huh7 cells was verified by RT-qPCR and western blotting. **(D,E)** Colony formation assays were performed to evaluate cell proliferation. **(F,G)** EdU assays for HCC cells were performed to evaluate cell proliferation. Scale bar, 50 μm. **(H)** Cellular growth curves were evaluated by CCK-8 assays in Huh7 cells. All data are presented as the means ± SD of three independent experiments. *p < 0.05, **p < 0.01, ***p < 0.001, and ****p < 0.0001.

To further evaluate the influence of the circCRIM1/miR-378a-3p/SKP2 axis on HCC cell growth, a short hairpin RNA and lentivirus were constructed to regulate the expression of SKP2. SKP2 was silenced or overexpressed in HCC cells, and the SKP2 expression was measured via qRT-PCR and western blotting (Figures 7A–C). Subsequently, colony formation, EdU, and CCK-8 assay results suggested that SKP2 overexpression accelerated the growth of Huh7 cells (Figures 7D–H). On the contrary, SKP2 silencing had an opposite effect on LM3 cells (Supplementary Figure S2). Furthermore, Skp2 overexpression largely reversed the inhibition of malignant biological behavior following disruption of circCRIM1 expression (Figures 7D–H), while the proliferation of HCC cells enhanced by circCRIM1 overexpression was inhibited by SKP2 knockdown (Supplementary Figure S2). In summary, circCRIM1 promotes HCC proliferation and relies on the miR-378a-3p/SKP2 axis.

As mentioned, circCRIM1 promotes cell proliferation by accelerating the transition from G1 to S phase. The SKP2 plasmid and si-circCRIM1 were transfected into HCC cells. The resulting data indicated that the G0/G1 phase arrest was reversed (Supplementary Figure S3A). Western blot assays were then used to detect key molecules involved in the G1/S transition. The related protein levels of CDK4, CDK6, cyclin D1, and cyclin E1 were significantly decreased, while P27 was upregulated in Huh7 cells after circCRIM1 silencing, which was consistent with the G0/G1 phase arrest (Supplementary Figure S3B). Previous research has reported that SKP2 is required for induction of vascular smooth muscle cell proliferation (Wang et al., 2017). We further examined whether circCRIM1 induces angiogenesis via SKP2 by performing rescue experiments. The results showed that culture with si-circCRIM1 CM, significantly inhibited HUVEC tube formation and endothelial recruitment compared with si-NC CM, which could be fully reversed by SKP2 overexpression (Supplementary Figures S3D,E). In summary, circCRIM1 promotes the angiogenesis of HUVECs and cell cycle transition of HCC cells via SKP2.

### CircCRIM1 Promotes HCC Xenograft Growth *in vivo*

**FIGURE 8:**
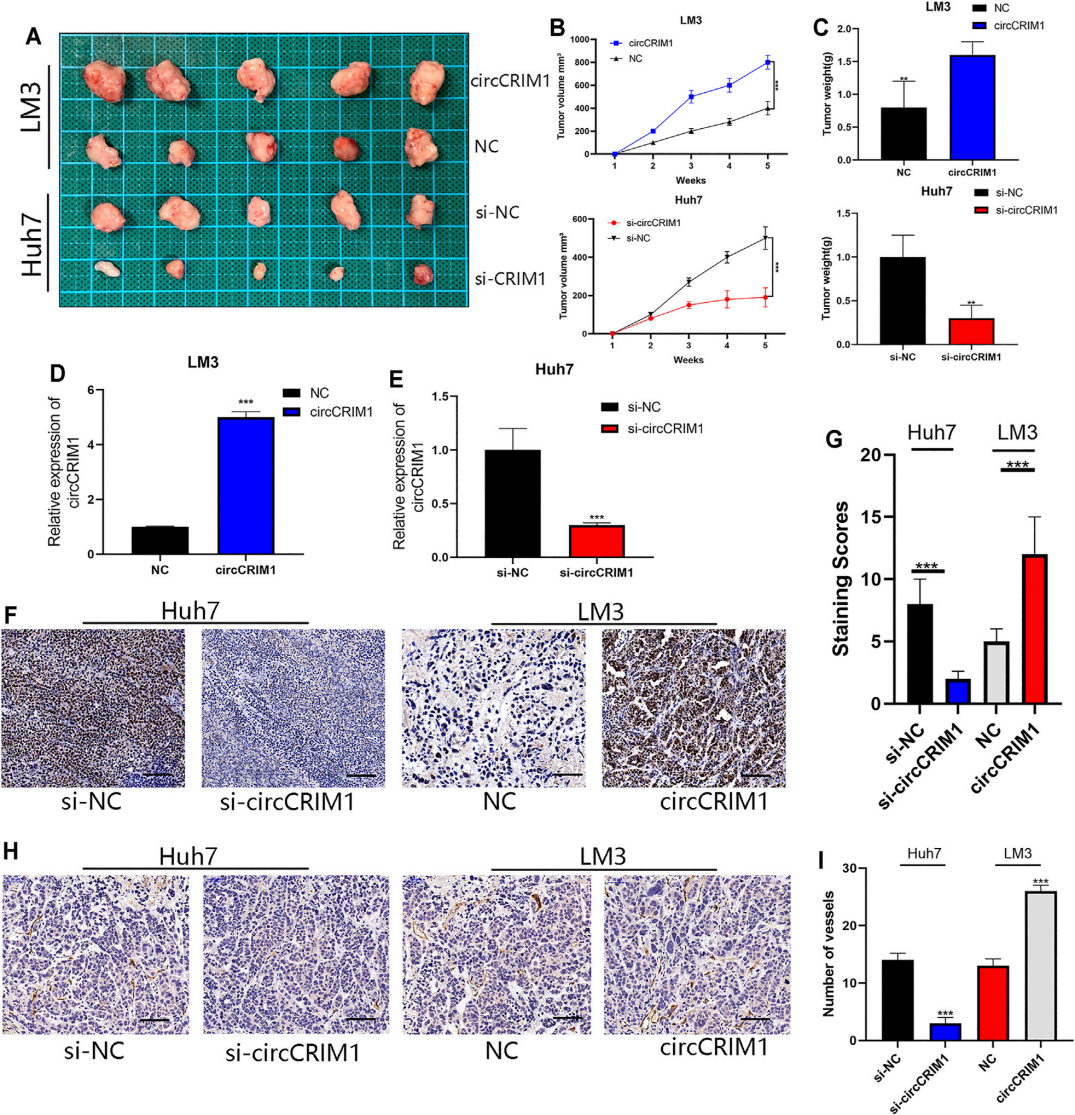
CircCRIM1 promotes HCC xenograft growth *in vivo*. **(A)** Representative images of subcutaneous xenograft tumors (*n* = 5 for each group). **(B)** Growth curves for tumor volumes, which were measured every week. **(C)** Tumor weight analysis. **(D,E)** Expression of circCRIM1 was observed in mouse tumors from each group. **(F,G)** IHC staining of xenograft tumors. SKP2 protein levels were analyzed based on IHC staining. Scale bar, 25 μm **(H,I)** Blood vessels were stained using anti-CD34 and positively stained vessels were counted in five areas per slide to determine maximum number of micro-vessels, ten slides per experiment. Scale bar, 25 μm. All data are presented as the means ± SD of three independent experiments. *p < 0.05, **p < 0.01, ***p < 0.001, and ****p < 0.0001.

To further investigate whether circCRIM1 contributes to the growth of tumor cells, an *in vivo* xenograft tumor model was established using LM3 and Huh7 cells. LM3 cells were effectively transfected with stably overexpressed circCRIM1 or NC and Huh7 cells were silenced by si-circCRIM1 or si-NC. Afterwards, the mice were evaluated every 3 days and sacrificed after 5 weeks. The results showed that tumors derived from si-circCRIM1 Huh7 cells grew more slowly than those derived from si-circCRIM1 NC cells (Figure 8A). Compared with the NC group, the si-circCRIM1 group developed tumors with lower weights and volumes (Figures 8B,C). In contrast, mice inoculated with LM3 circCRIM1-overexpressing cells had a larger mean tumor volume and weight than the control group during the fifth week (Figures 8A–C). Additionally, circCRIM1 expression was observed in the tumors of mice from each group (Figures 8D,E). IHC was also used to investigate Skp2 level and CD34 expressions in xenografts. Lower expression of SKP2 in the si-circCRIM1 group and higher SKP2 expression in the circCRIM1 group were detected (Figures 8F,G). Compared with the si-NC tumors, the tumor-containing si-circCRIM1 Huh7 cells had smaller and fewer vessels. The CD34-marked tumor vessels in circCRIM1-overexpressing tumors were larger and more abundant, while SKP2 expression was obviously higher (Figures 8H,I). Overall, circCRIM1 promoted the proliferation and angiogenesis of HCC *in vivo*, confirming our *in vitro* findings*.*

## Discussion

Statistical analysis shows that although diagnosis and treatment methods have greatly improved during the past decades, the five-year OS rate in HCC patients remained at a disappointing level (Allemani et al., 2018). Currently, chemotherapy, targeted therapy, and immunotherapy are the primary treatment choices for advanced HCC patients (Greten et al., 2019; Mahipal et al., 2019). However, surgery is still the most efficient tool that prolongs patient survival (Bai et al., 2021). Therefore, it is necessary to investigate new molecular HCC targets for early diagnosis and treatment.

Recently, circRNAs have been demonstrated to play critical roles in the progression of cancers, such as migration, invasion, metastasis (Hu et al., 2020), and chemotherapy resistance (Weng et al., 2021). CircRNAs regulate target gene expression by sponging miRNAs, directly binding to specific proteins, or encoding functional oligopeptides (Wu et al., 2020; Zhou et al., 2021). For example, circZNF566 sponged miR-4738-3p to accelerate the progression of HCC(Li S. et al., 2020). CircBACH1 promoted the growth of HCC by interacting with p27 (Liu B. et al., 2020). In colon cancer, circFNDC3B encoded a novel oligopeptide to regulate cancer progression. CircCRIM1 has been identified as a tumor-associated factor in human cancers. For example, in nasopharyngeal carcinoma, circCRIM1 has been shown to promote the proliferation, invasion, metastasis, and chemoresistance by sponging miR-422a (Hong et al., 2020), while circCRIM1 accelerated the progression and facilitated the autophagy of osteosarcoma by targeting miR-432-5p (Liu J. et al., 2020). However, circCRIM1 has also been identified as a tumor-suppressive factor that inhibits metastasis in lung adenocarcinoma by interacting with miR-182 and miR-93 (Hong et al., 2020). The present study determined that circCRIM1 has a high expression in HCC samples and is closely related to malignant HCC progression, making it a promising prognostic biomarker. Tumor growth slows down when tumor volume exceeds 2–3 mm^3^ (Harper and Moses, 2006) due to the lack of a vascular system. In this case, the angiogenic switch is activated to facilitate cancer growth and metastasis. Importantly, it has been discovered that circCRIM1 silencing significantly inhibits both HCC cell proliferation and angiogenesis, indicating that circCRIM1 has a strong tumor-promoting effect. Emerging evidence suggests that circRNAs can function as a sponge to silence miRNA and regulate expression of the targeted gene (Han et al., 2017; Wu et al., 2021).

Using bioinformatics analysis with miRanda, circbank, and RNAhybrid, miR-378a-3p was determined to be a target of circCRIM1 in HCC cells and identified as a tumor-suppressive factor. Cai et al. have reported that it promotes the sorafenib sensitivity in HCC cells (Lin et al., 2020). Moreover, Cheng et al. have suggested that the microRNA inhibition accelerates the metastasis of oral squamous cell carcinoma (Wang et al., 2018). In addition, Han et al. have observed that it inhibits the growth of colorectal cancer and induces apoptosis (Li H. et al., 2014). The present RNA pull-down assay results revealed that it is the most abundant miRNA in the complex. In addition, dual-luciferase reporter assay, FISH, and anti-AGO2 RNA immunoprecipitation (RIP) assays collectively suggested HCC cell cytoplasm might be the location of circCRIM1 and miR-378a-3p, where circCRIM1 interaction occurs and decreases its expression. Furthermore, rescue experiments revealed that circCRIM1 knockdown effects can be reversed by the inhibitors. The present experimental data demonstrated that circCRIM1 sponge this miRNA during the progression of HCC, which is consistent with the results mentioned above, indicating that circCRIM1 plays a critical role in HCC progression.

SKP2 is a RING-finger type ubiquitin ligase, a well-characterized member of CRLs, and a functional component of ligase complex SCF. Several statistical analyses showed that SKP2 promotes the progression and induces tumorigenesis of nasopharyngeal carcinoma (Yu et al., 2019) and accelerates the growth of renal cell carcinoma (Chen et al., 2021). The present experimental results verified SKP2 as the most likely target of miR-378a-3p. Furthermore, the microRNA reversed the effect of circCRIM1 by promoting SKP2 expression and HCC proliferation, while SKP2 silencing attenuated the tumor-promoting capability of circCRIM1. This result revealed that circCRIM1 can sponge it to increase the expression of target gene SKP2, thus promoting the progression of HCC. Subsequently, circCRIM1 was shown to promote HCC angiogenesis *in vivo* and *in vitro* via the circCRIM1/miR-378a-3p/SKP2 axis. All of these results provided a promising direction for circCRM1-based biotherapeutics. Several studies have suggested that circRNAs exist in the serum or some tumor tissues (Tian and Wang, 2016), suggesting that they have the potential to be biomarkers for specific diseases or targets for carcinoma treatment. Thus, more studies are needed to verify its presence in the serum of HCC patients. In addition, the function and mechanism of circCRIM1 in HCC need to be determined and its potential to be a prognosis target explored.

In summary, the present study revealed that circCRIM1 is highly upregulated in HCC tissues and acts as a promoter in tumorigenesis and angiogenesis via the circCRIM1/miR-378a-3p/SKP2 axis (Supplementary Figure S3C). The performance of circCRIM1 *in vivo* indicates that it can function as a possible prognosis prediction factor and used for future targeted therapy in HCC patients.

## Data Availability

The raw data supporting the conclusion of this article will be made available by the authors, without undue reservation.

## Supplementary Material

Supplementary Figure S1 MiR-378a-3p attenuates the oncogenic effects of circCRIM1 in LM3 cells.

Supplementary Figure S2 CircCRIM1 promotes LM3 cell proliferation via the miR-378a-3p/SKP2 axis.

Supplementary Figure S3 CircCRIM1 promotes HCC proliferation and angiogenesis *via* the miR-378a- 3p/SKP2 axis.

Supplementary Table S1 Primers for qRT-PCR used in this study.

Supplementary Table S2 Antibodies used in this study.

Supplementary Table S3 Eight candidate miRNAs binding to circCRIM1 predicted by cross-analyzing three miRNA target prediction databases.

Supplementary Table S4 Twenty-one candidate miRNAs binding to miR-378a-3p predicted by cross-analyzing four mRNA target prediction databases.
