# Supplementary material for: CircCRIM1 Promotes Hepatocellular Carcinoma Proliferation and Angiogenesis by Sponging miR-378a-3p and Regulating SKP2 Expression
Source: Front Cell Dev Biol. 2021 Nov 12;9:796686. doi: 10.3389/fcell.2021.796686 (PMC8634842; doi:10.3389/fcell.2021.796686)
Supplement: Supplementary file 2 [file Table1.docx]

| **Table S1** | |
| --- | --- |
| **PCR** **PRIMERS** |  |
| ZBTB20 | Forward: 5’-CGAGCGCATTCACAGCATCAACCTT-3’  Revers: 5’-TCTCGATGTCGCTGTAGCCAAGCAG-3’ |
| DSCAM | Forward: 5’-ACCACAACAACAACAACAG-3’  Reverse: 5’-ATGATGAGACCAGAACTTCC-3’ |
| PLAGL2 | Forward: 5’-CAGAGACCATATAGCTGCCC-3’  Reverse: 5’-CCTTGCGGTGAAACATCTTATC-3’ |
| IL6ST | Forward: 5’-GGCTTGCCTCCTGAAAAACC-3’  Reverse: 5’-ACTTCTCTGTTGCCCACTCAG-3’ |
| TMED7-TICAM2 | Forward: 5’-TCGAG-CGGGCCGCCCGGGCAGGT-3’  Reverse: 5’-AGCGG-GTGGTCGCGGCCGAGGT-3’ |
| SKP2 | Forward：5’-CCA GGA GAT TCC AGA CCTGAG T-3’  Reverse：5’-TGT CAC TCC CTT TGC TCT TCAG-3’ |
| QSER1 | Forward: 5’-CAAAGGGGGTGTCCTCTATG-3’  Reverse: 5’-GTTGCTCGTCCTACATGTGC-3’ |
| CAMKK2 | Forward: 5’-CGTTGAGATCCTCGGCTACTG-3’ Reverse: 5’-GGGTCCTTCAGGTTCTGTTCC-3’ |
| CBX5 | Forward: 5’-GCAGACGTTAGCGTGAGTG-3’  Reverse: 5’-AGACAGCACAATAACCAGCAC-3’ |
| KPNA6 | Forward: 5’-GGCACTGGGAAACATAGCTGG-3’  Reverse: 5’-CGTGTGGACTTGGTAAGGAGT-3’ |
| SOBP | Forward: 5’-CGCCAGCACATTTCTGTTCTC-3’  Reverse: 5’-AGCCCTGGGTAGCTT GTGTTT-3’. |
| REST | Forward: 5’-ACTCAGCGTCGTAGAACCTCA-3’  Reverse: 5’-CGAAAGGGTTTGGTCTTCGAG-3’ |
| MAP2K6 | Forward: 5’-GTCCA TTCAC CGTGA CCTTC TA-3’  Reverse: 5’-GACGT CTCGA TGGAT AACGA ACA-3’ |
| DACT1 | Forward: 5’-GGA AGA GGA CAG GCT TGG AAAC-3’ Reverse: 5’-GTC CCA TTG TTC AGA GAA GGT ATC -3’ |
| YY1 | Forward: 5’-AGCAGAAGCAGGTGCAGATCAA-3’  Reverse: 5’-CTGCCAGTTGTTTGGGATCT-3’ |
| TMEM245 | Forward: 5’-CAAGCGTGTCCGTAAGGATT-3’  Reverse: 5’-ACAATTGGGTTCTGCAGCTT-3’ |
| DYRK1 | Forward: 5’-TCTGGGTATTCCACCTGCTC-3’  Reverse: 5’-GTCCTCCTGTTTCCACTCCA-3’ |
| SHANK3 | Forward 5’-ACCTTCATCCACCCACTCAC-3’  Reverse 5’-GGGCCTGTACATCCACAAAC-3’ |
| ATG12 | Forward 5’-CTTAAACTGGTGGCCTCGGA-3’  Reverse 5’-TTTCTGGCTCATCCCCATGC-3’ |
| PAG1 | Forward: 5’-GCAGCGGACAGATG-CAGAT-3’  Reverse: 5’-CAGGAAGATGAG-GAAGGTGATGA-3’ |
| ZNF652 | Forward: 5’-GTCTCAGGTCAGATCGAGAACA-3’  Reverse: 5’-ACTCTGTGTCCATACTGATGCC-3’ |
| GAPDH | Forward: 5’-GTGAAGCAGGCGTCGGA-3’  Reverse: 5’-AGCCCCAGCGTCAAAGG-3’ |
| GAPDH (divergent) | Forward: 5’-TGTACCATCAATAAAGTACCCTGTG-3’  Reverse: 5’-AAATCCGTTGACTCCGACCT-3’ |
| U6 | Forward: 5’-CTCGCTTCGGCAGCACA-3’  Reverse: 5’-AACGCTTCACGAATTTGCGT-3’ |
| CircCRIM1 | Forward: 5’-CTGTACTTTGCCAACAAGATGAGA-3’  Reverse: 5’-GCACATATCCTGACTTGGAAACTC-3’ |
| miR-665 | Forward: 5’-GGTGAACCAGGAGGCTGAGG-3’  Reverse: 5’-CAGTGCAGGGTCCGAGGTAT-3’ |
| miR-146a-5p | Forward: 5’-CGCGTGAGAACTGAATTCCA-3’  Reverse: 5’-AGTGCAGGGTCCGAGGTATT-3’ |
| miR-422a | Forward: 5’-ACUGGACUUAGGGUCAGAAGGC-3’ Reverse: 5’-GCCUUCUGACCCUAAGUCCAGU-3’ |
| miR-378h | Forward: 5’-GGGACTG-GACTTGGTGTC-3’  Reverse: 5’-CAGTGCGTGTCGTGGAGT-3’ |
| miR-378a-3p | Forward:5’- CGCGACTGGACTTGGAGTCA -3’  Reverse:5’- AGTGCAGGGTCCGAGGTATT -3’ |
| miR-4436a | Forward:5’-AACAGAGCAGGACAGGCAGAA-3'  Reverse:5’-GTCACGTCCCAGGCTCCA-3' |
| miR-4761-3p | Forward:5’-ATGCGCGCAAATTCATGTTCAA-3'  Reverse:5’-GTCACGTCCCAGGCTCCA-3' |
| miR-5195-3p | Forward:5’-GCCTGTAGGCATCATCGCCAG-3’  Reverse:5’- GATAGAGTGACGTGAAGTAG-3’ |
| **si-RNAs** |  |
| MiR-378a-3p mimics | 5’-ACUGGACUUGGAGUCAGAAGGC-3’ |
| MiR-378a-3p inhibitor | 5’-GCCUUCUGACUCCAAGUCCAGU-3’ |
| Si-circCRIM1 | 5’-GCCAACAAGAUGAGAACUGTT-3’ |
| Shskp2 | 5'-CCTTAGACCTCACAGGTAA-3' |
