# Supplementary figures and images for: CircCRIM1 Promotes Hepatocellular Carcinoma Proliferation and Angiogenesis by Sponging miR-378a-3p and Regulating SKP2 Expression
Source: Front Cell Dev Biol. 2021 Nov 12;9:796686. doi: 10.3389/fcell.2021.796686 (PMC8634842; doi:10.3389/fcell.2021.796686)

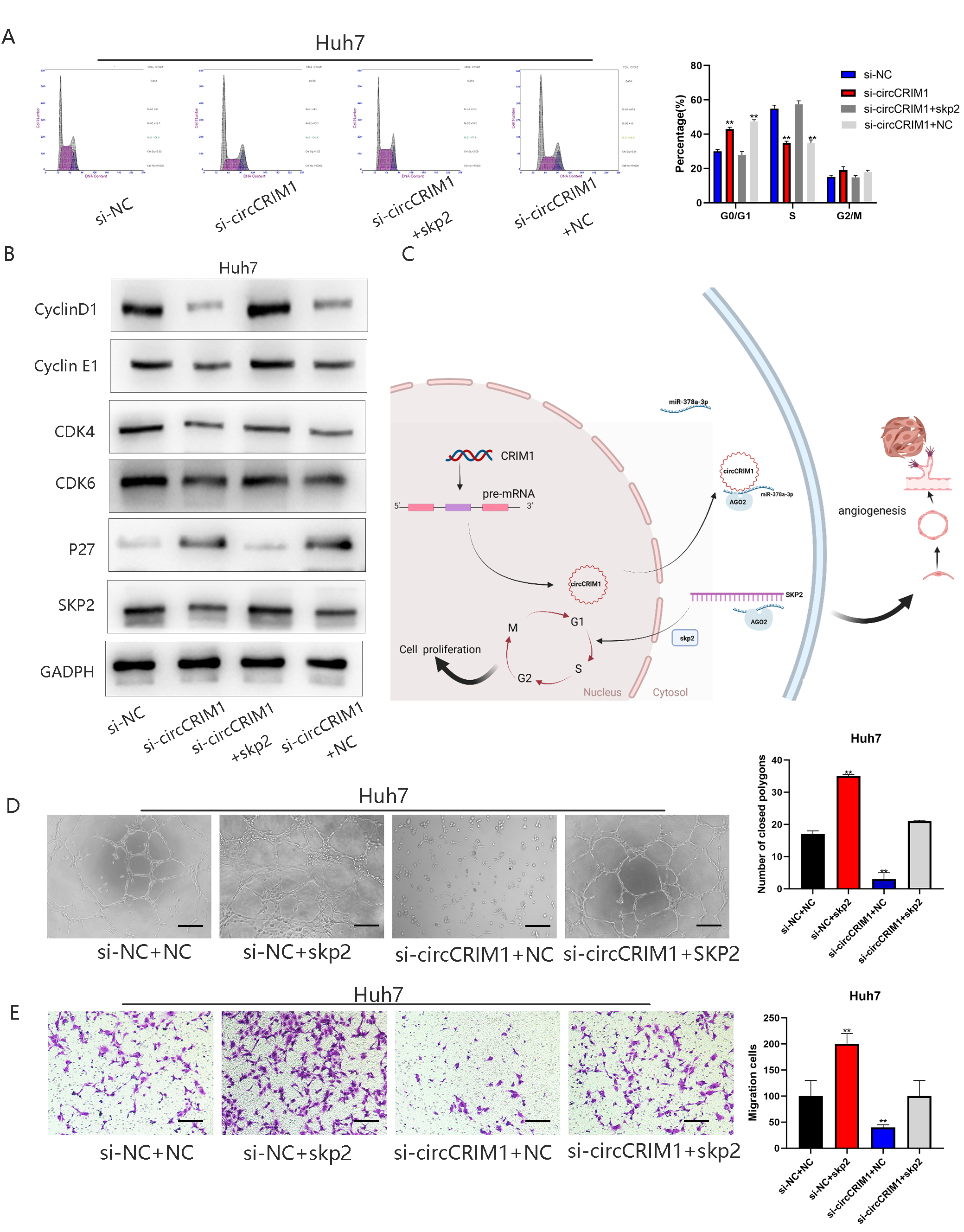

Supplement: Supplementary file 3 [file Image3.TIF]

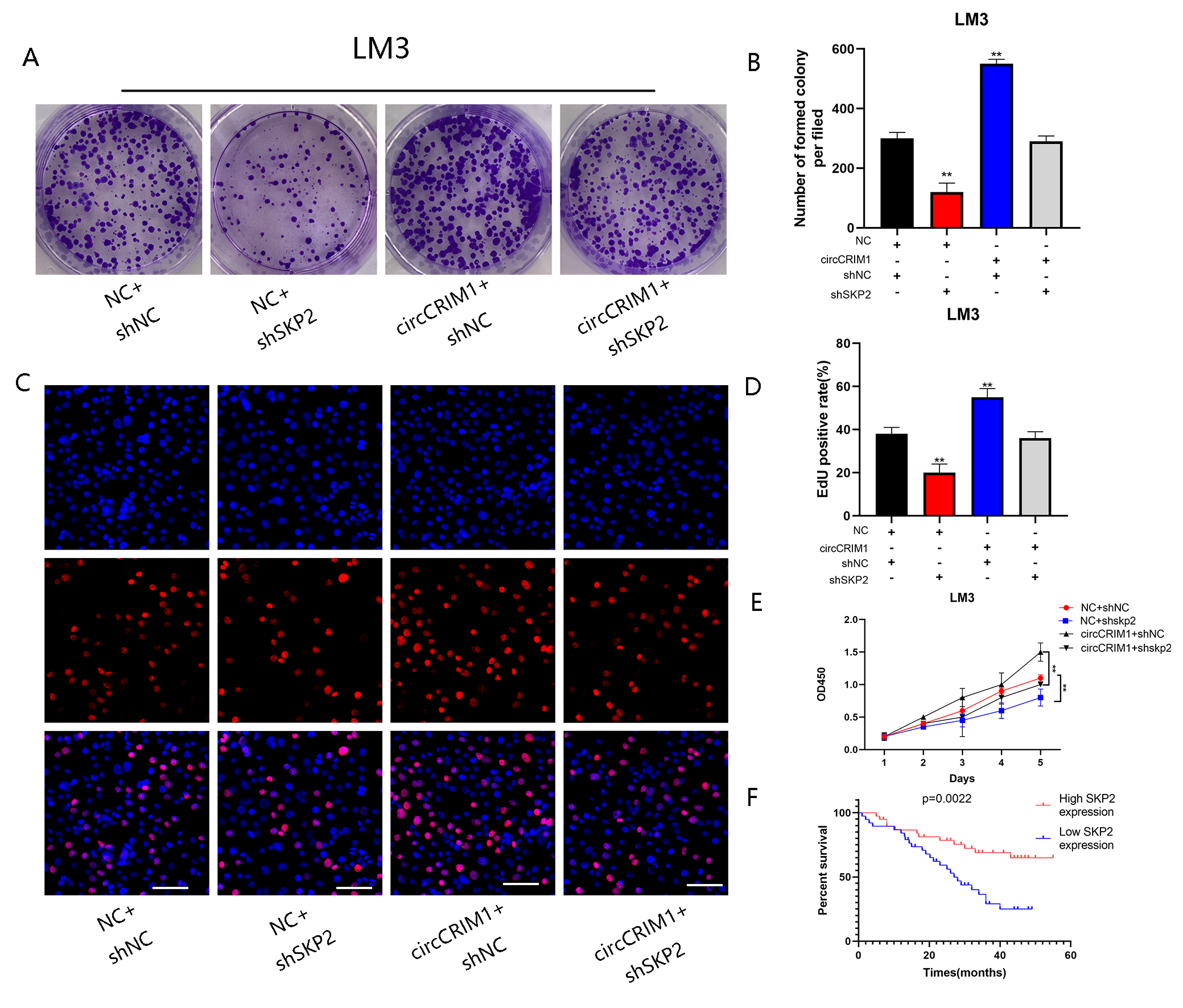

Supplement: Supplementary file 4 [file Image2.TIF]

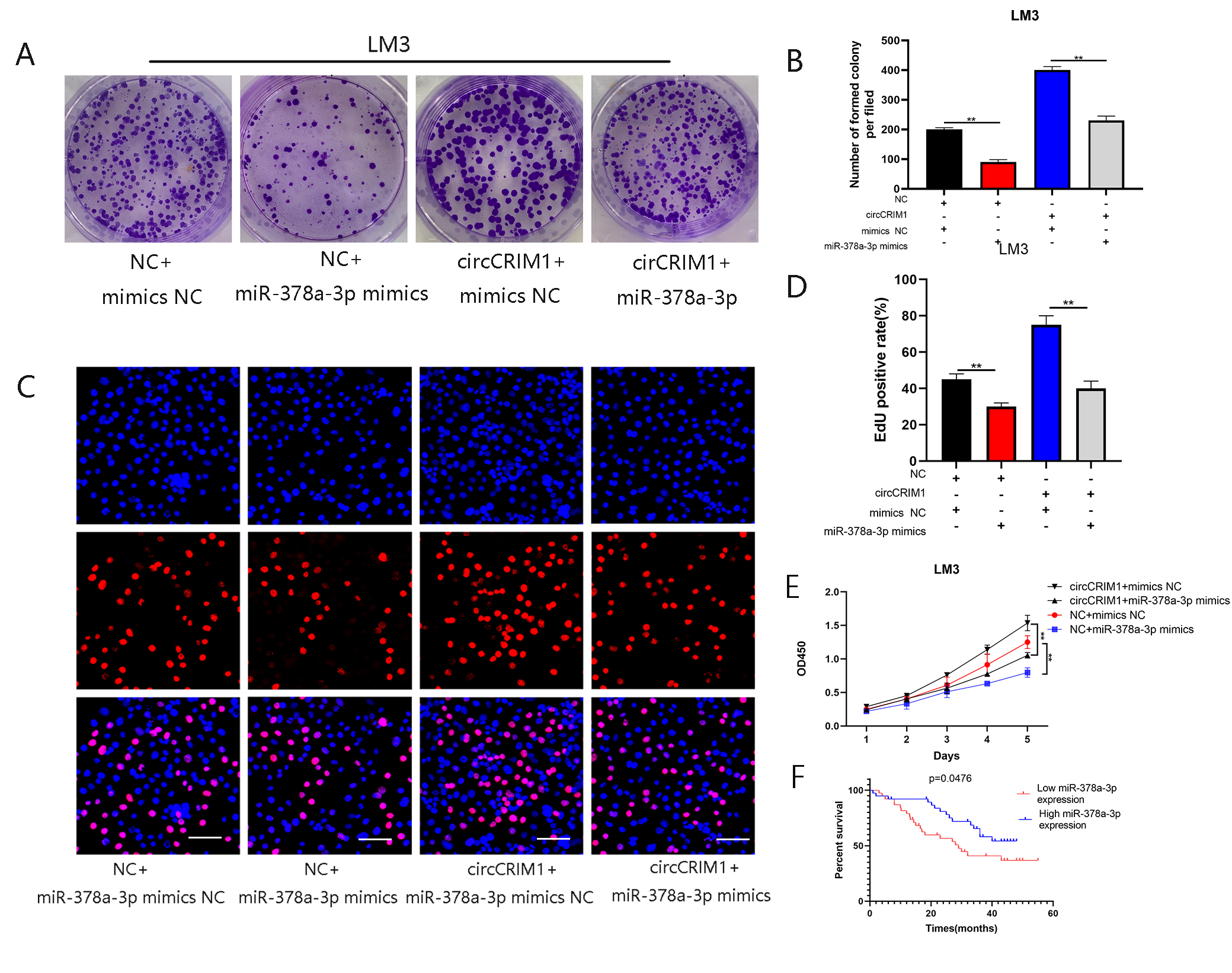

Supplement: Supplementary file 6 [file Image1.TIF]
