# Supplementary material for: CircCRIM1 Promotes Hepatocellular Carcinoma Proliferation and Angiogenesis by Sponging miR-378a-3p and Regulating SKP2 Expression
Source: Front Cell Dev Biol. 2021 Nov 12;9:796686. doi: 10.3389/fcell.2021.796686 (PMC8634842; doi:10.3389/fcell.2021.796686)
Supplement: Supplementary file 7 [file Table2.docx]

**TableS2. Antibodies used in this study**

| Cyclin D1 | Cell signaling Technology | 2978S |
| --- | --- | --- |
| Cyclin E1 | Cell signaling Technology | 20808S |
| CDK4 | Cell signaling Technology | 12790S |
| Skp2 | Proteintech | 15010-1-AP |
| P27 | Cell signaling Technology | 3686T |
| GADPH | Cell signaling Technology | 5174T |
| CDK6 | Cell signaling Technology | 13331S |
| CD34 | Cell signaling Technology | 3569S |
| **Secondary antibody** |  |  |
| anti-rabbit IgG-HRP | Proteintech | SA00001-15 |
| anti-mouse IgG-HRP | Proteintech | SA00001-1 |
| **RIP:** |  |  |
| anti-Argonaute-2 | Abcam | ab186733 |
| Skp2 | Proteintech | 15010-1-AP |
| IgG | Abcam | ab172730 |
